# Evolution and Phylogeny of Large DNA Viruses, *Mimiviridae* and *Phycodnaviridae* Including Newly Characterized *Heterosigma akashiwo Viru*s

**DOI:** 10.3389/fmicb.2016.01942

**Published:** 2016-11-30

**Authors:** Fumito Maruyama, Shoko Ueki

**Affiliations:** ^1^Department of Microbiology, Graduate School of Medicine, Kyoto UniversityKyoto, Japan; ^2^Institute of Plant Science and Resources, Okayama UniversityKurashiki, Japan

**Keywords:** nucleocytoplasmic large DNA virus, *Heterosigma akashiwo virus*, *Phycodnaviridae*, *Mimiviridae*, evolution, phylogeny

## Abstract

Nucleocytoplasmic DNA viruses are a large group of viruses that harbor double-stranded DNA genomes with sizes of several 100 kbp, challenging the traditional concept of viruses as small, simple ‘organisms at the edge of life.’ The most intriguing questions about them may be their origin and evolution, which have yielded the variety we see today. Specifically, the phyletic relationship between two giant dsDNA virus families that are presumed to be close, *Mimiviridae*, which infect *Acanthamoeba*, and *Phycodnaviridae*, which infect algae, is still obscure and needs to be clarified by in-depth analysis. Here, we studied *Mimiviridae*–*Phycodnaviridae* phylogeny including the newly identified *Heterosigma akashiwo virus* strain *HaV53*. Gene-to-gene comparison of *HaV53* with other giant dsDNA viruses showed that only a small proportion of *HaV53* genes show similarities with the others, revealing its uniqueness among *Phycodnaviridae*. Phylogenetic/genomic analysis of *Phycodnaviridae* including *HaV53* revealed that the family can be classified into four distinctive subfamilies, namely, Megaviridae (Mimivirus-like), *Chlorovirus*-type, and *Coccolitho*/*Phaeovirus*-type groups, and *HaV53* independent of the other three groups. Several orthologs found in specific subfamilies while absent from the others were identified, providing potential family marker genes. Finally, reconstruction of the evolutionary history of *Phycodnaviridae* and *Mimiviridae* revealed that these viruses are descended from a common ancestor with a small set of genes and reached their current diversity by differentially acquiring gene sets during the course of evolution. Our study illustrates the phylogeny and evolution of *Mimiviridae*–*Phycodnaviridae* and proposes classifications that better represent phyletic relationships among the family members.

## Introduction

Nucleocytoplasmic large DNA viruses (NCLDVs) possess double-stranded DNA (dsDNA) genomes with sizes in some cases over 2 Mbp, and particle sizes that can exceed 1 μm. The discovery of NCLDVs challenges the traditional concept of a virus as an obligate pathogen with small, ‘filterable’ particle size and a simple genome, and expands the biological definition of a virus (Sharma et al., 2016). Their large genomes suggest that their life cycles, i.e., infection processes in their hosts, are complex (Fischer and Sansom, 2002; Gazzarrini et al., 2003; Chen et al., 2005; Frohns et al., 2006; Claverie and Abergel, 2010; Hamacher et al., 2012; Kuznetsov et al., 2013; Mutsafi et al., 2013; Chelikani et al., 2014). Further discoveries of NCLDVs that continue to set records for genome size have ignited discussion about their origin and evolution (Iyer et al., 2006; Suzan-Monti et al., 2006; Koonin and Yutin, 2010; Arslan et al., 2011; Colson et al., 2011; Abrahao et al., 2014; Abergel et al., 2015).

*Heterosigma akashiwo virus* (*HaV*) is an NCLDV isolated from a unicellular alga, *Heterosigma akashiwo. H. akashiwo* is a eukaryotic alga that is widely distributed in temperate, neritic waters, including off the coasts of North and South America, Eastern Asia, Oceania, and the Northern Atlantic region (Lackey and Lackey, 1963; Rojas de Mendiola, 1979; Rensel et al., 1989; Chang et al., 1990; Mackenzie, 1991; Honjo, 1993; Taylor, 1993; Tseng et al., 1993; O’Halloran et al., 2006). It is a member of class *Raphidophyceae*, and is a bloom forming species. Like *Aureococcus anophagefferens virus* (*AaV*) (Moniruzzaman et al., 2014), *Emiliania huxleyi virus* 86 (*EhV*) (Schroeder et al., 2002; Wilson et al., 2005), and Phaeocystis globosa virus (PgV) (Brussaard et al., 2004; Baudoux and Brussaard, 2005), *HaV* was originally isolated as a bloom terminating factor (Nagasaki and Yamaguchi, 1997) and is of ecological importance in controlling algal populations in nature. A *HaV* strain, *HaV01*, was characterized as a dsDNA virus with a genome size of ∼290 kbp (Nagasaki et al., 2005), and we recently reported the complete genome sequence of *HaV* strain 53 (*HaV53*, submitted for publication).

Nucleocytoplasmic large DNA viruses infecting marine algae as natural hosts, including *HaV53*, have been collectively classified as a family, *Phycodnaviridae*, with prefix ‘phyco-’ meaning algae. The term ‘algae’ is rather broad and includes both multi- and unicellular, brown and green, aquatic photosynthetic organisms. Presumably reflecting the diversity of host species, several past studies have suggested that *Phycodnaviridae* consists of groups of viruses with different features. Notably, while classified as phycodnaviruses, Chrysochromulina ericina virus (CeV), PgV and AaV are similar in many respects to *Mimiviridae* (Brussaard et al., 2004; Monier et al., 2008; Moniruzzaman et al., 2014), giant NCLDVs that infect *Acanthamoeba* (Raoult et al., 2004, 2007; Claverie et al., 2006, 2009; Raoult and Forterre, 2008), rather than to other phycodnaviruses. Several studies strongly suggest that these three viruses are closely related to *Mimivirus* (Yutin et al., 2009, 2013, 2014; Fischer et al., 2010; Thomas et al., 2011; Santini et al., 2013; Moniruzzaman et al., 2014; Legendre et al., 2015). The terms ‘extended *Mimivirus*’ and ‘Megaviridae’ have been used in several studies to encompass the *Mimivirus* lineages, smaller *Mimiviruses* (i.e., *Cafeteria roenbergensis virus*, *CroV*), and phycodnaviruses that share characteristic features with *Mimiviruses*, although ‘Megaviridae’ is yet to be adopted by the International Committee for Taxonomy of Viruses (ICTV) as a family classification. Collectively, the current family *Phycodnaviridae*, officially adopted by ICTV, includes viruses that may not necessarily be closely related evolutionarily or phylogenetically.

While the origin and evolutionary history of NCLDVs in general are of great interest, the diversity of NCLDVs imposes difficulties in collectively evaluating their phylogenetic relationships. Attempts to classify NCLDVs and to infer their evolutionary history have involved comparisons of their sequences and genomic compositions. Orthologous genes in NCLDVs can be identified by direct comparisons of viral genes, or using the established Nucleo-Cytoplasmic Virus Orthologous Groups (NCVOGs) database (Yutin et al., 2009). In this study, we analyzed the phylogenetic relatedness of *HaV53* to members of the *Phycodnaviridae* and *Mimiviridae*. Our results underscore the validity of demands for the reclassification of the current *Phycodnaviridae* family, in addition to providing insights into the evolution of *Mimiviridae* and *Phycodnaviridae* including *HaV53*.

## Materials and Methods

### Sequence Information and Database

Viral genomes and encoded amino acid sequences were downloaded from ftp://anonymous@ftp.ncbi.nlm.nih.gov/genomes/Viruses/. The NCVOG database was downloaded from ftp://ftp.ncbi.nih.gov/pub/wolf/COGs/NCVOG/.

Amino acid sequences coded by genomes of following viruses were incorporated into the NCLDV database used in this study; *HaV53*, *Aureococcus anophagefferens virus* MM 2014 (*AaV*, NC024697), *Acanthocystis turfacea Chlorella virus* 1, (*AtCV*, NC008724), Bathycoccus RCC1105 virus (BpV, NC014765), Haptolina ericina virus CeV-01B (formerly Chrysochromulina ericina virus, CeV, KT820662), *Cafeteria roenbergensis virus* (*CroV*, NC014637), *Emiliania huxleyi virus86* (*EhV*, NC007346), *Ectocarpus siliculosus virus1* (*EsV*, NC002687), Feldmannia species virus (FsV, NC011183), Megavirus chilensis (MegaV, NC016072), *Acanthamoeba polyphaga mimivirus* (*MimiV*, NC014649), *Acanthamoeba polyphaga moumouvirus* (*MoumouV*, NC020104.1), *Micromonas RCC1109 virus* (*MpV*, NC014767), Ostreococcus lucimarinus virus 5 (OlV5, NC020852), *Ostreococcus tauri virus 1*, (*OtV1*, NC013288), *Ostreococcus tauri virus 5* (*OtV5*, NC010191), *Paramecium bursaria Chlorella virus 1* (*PBCV1*, NC000852), Phaeocystis globosa virus (PgV, NC021312), *Autographa californica nucleopolyhedrovirus* (AcNPV, NC 001623), *African swine fever virus* (*ASFV*, NC 001659), *Melanoplus sanguinipes entomopoxvirus* (*MsEV*, NC 001993), *Amsacta moorei entomopoxvirus* (*AEPV*, NC 002520), *Culex nigripalpus NPV* (*CnNPV*, NC 003084), *Heliothis virescens ascovirus 3e* (HvaV, NC_009233), *Infectious spleen and kidney necrosis virus* (*ISKNV*, NC 003494), *Mamestra configurata NPV-A* (*McNPV*, NC 003529), *Lymphocystis disease virus china* (*LDV*, NC 005902), *Spodoptera litura granulovirus* (*SlGV*, NC 009503), *Marseillevirus* (*MarV*, NC 013756), *Rodent herpesvirus Peru* (*RHV*, NC 015049), *Lausannevirus* (*LausV*, NC 015326), *Wiseana iridescent virus* (*WiV*, NC 015780), Pithovirus sibericum (PithoV, NC 023423), Pandravirus dulces (PandraV, NC_021858), Mollivirus (MolliV, NC 027867), and *Human herpesvirus 3* (*HHV3*, NC 001348).

For NCLDV CP (NCVOG0022), D5-like helicase primase (NCVOG0023), and DNA polymerase B (NCVOG0038) phylogenetic analyses (**Supplementary Figure S2**), the orthologs were determined by choosing best-hit target sequences obtained by BLASTP search (*E*-value < 10^-20^) using the NCVOG orthologs as queries, and the databases were created from the amino acid sequences coded by the genomes of above mentioned viruses and of *Ambystoma tigrinum virus* (*AtV*, NC_005832), *Bovine papular stomatitis virus* (*BpsV*, NC_005337), *Fowlpox virus* (*FpV*, NC_002188), *Frog virus* (*FrogV*, NC_005946), *Invertebrate iridescent virus 6* (*InvIV6*, NC_003038), *Lymphocystis disease virus* 1 (*LDV1*, NC_001824), *Molluscum contagiosum virus subtype 1* (*McV*, NC_001731), *Sheeppox virus* (*ShpV*, NC_004002), *Singapore grouper iridovirus* (*SgiV*, NC_006549), *Swinepox virus* (*SwpV*, NC_003389), *Trichoplusia ni ascovirus* (*TnaV*, NC_008518), *Vaccina virus* (*VaccinaV*, NC_006998), and Yaba monkey tumor virus (*YmtV*, NC_005179).

### Software

BLAST+ (version 2.2.31) executables were downloaded from ftp://ftp.ncbi.nlm.nih.gov/blast/executables/blast+/LATEST/. Databases for BLASTP and PSI-BLAST searches were constructed according to the provided manual. For phyletic studies, 18 viruses, namely AaV, *AtCV*, BpV, CeV, *CroV*, *EhV86*, *EsV*, FsV, *HaV53*, MegaV, *MimiV*, *MoumouV*, *MpV1*, OlV5, *OtV1*, *OtV5*, *PBCV1*, and PgV were selected. Proteins equal to or larger than 100 aa encoded by each virus were extracted and used as queries. When a single open reading frames (ORF) hit multiple target sequences in databases, the hit with the highest bit score was selected for further study. Similarly, when multiple ORFs in a viral genome hit the same target sequence in NCVOG, the ORF that hit with the highest bit score was selected for further study to identify a true ortholog rather than paralogs.

Multiple sequence alignments and phylogenetic reconstructions by neighbor-joining were performed in ClustalX version 2.1 (Larkin et al., 2007). Poorly conserved regions and positions including gaps were removed prior to phylogenetic analysis. Neighbor-joining phylogenetic inferences were conducted, and the confidence of the branching was assessed using 1,000 bootstrap resampling replicates of the analyzed dataset.

Pan-genome analysis was conducted using PGAP software using cut-off values of 20% identity and *E*-value < 10^-5^ (Zhao et al., 2012). In this analysis, orthologs in each virus in the dataset were determined by all-to-all BLASTP search followed by MCL, and phyletic inference calculated by neighbor-joining based on the presence/absence matrix of the orthologs in each combination of the viruses (Zhao et al., 2012).

Gain and loss of gene families during evolution was mapped on a guide tree based on the concatenated sequence of nine preserved genes (**Figure 4A**) using COUNT software (Csuros, 2010; Kamneva and Ward, 2014). For each gene family, Wagner parsimony with gene gain penalties of 1 and 5 were used to infer the most parsimonious ancestral gene sets with different gain/loss pressures. We chose Wagner parsimony, rather than other protocols, because it allows multiple gains with penalties and infers gene family expansion and contraction (Csuros, 2010). For both PGAP and COUNT analyses, we selected genes coding for proteins with 100 aa or more. The resulting trees from all the analyses were visualized using Geneious 9.0.5.

## Results

### *HaV53* Genes and NCLDV Orthologs

**FIGURE 1:**
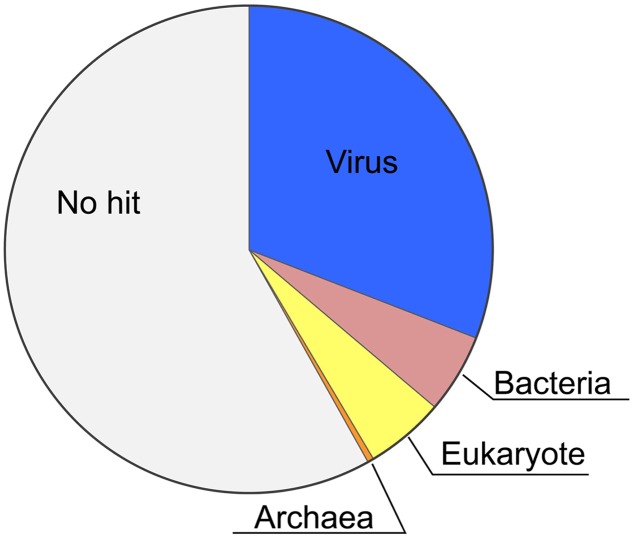
**Phyletic distribution of *HaV53* gene homologs.** The best-hit homolog in the NCBI NR database to the each *HaV53* open reading frames (ORF) was determined by BLASTP (*E*-value < 10^-5^), and source organisms were identified.

Recently, we completed sequencing of the *HaV53* genome (GenBank accession number KX008963, (Ogura et al., 2016). To gain further insight into the potential functions of *HaV53* gene products, we predicted the functions of *HaV53* ORF using the NCVOG database (Yutin et al., 2009, 2014) and the NCBI NR protein database. *HaV53* genes were annotated by searching the databases using BLASTP with E-value cutoff set to 10^-5^ (**Supplementary Figure S1**; **Supplementary Table S1**). All the search results were further supported by PSI-BLAST results (*E*-value < 10^-8^, three iterations), confirming homology to the target sequences. Seventy-four *HaV53* ORFs exhibited similarities to NCVOGs, while for 27, the best-hit proteins were from bacterial, archaeal, and eukaryotic genomes (**Supplementary Table S1**; **Figure 1**). The remaining *HaV53* ORFs did not exhibit significant similarity to any protein in the databases (**Figure 1**). As expected, *HaV53* possesses orthologs of four previously sequenced *HaV01* genes, AGB-1, UKCH-2, NCLDV major capsid protein, and B-family DNA polymerase, with high sequence similarities (**Supplementary Table S1**). Like other NCLDVs, multiple occurrences of genes annotated with identical functions were also observed within the subset of *HaV53* ORFs. To evaluate if these genes were originated from insertion of multiple homologous genes from different source organisms, or due to duplication of a gene acquired from single horizontal gene transfer, we evaluated the homologies among the *HaV53* ORFs (paralogs) and compared their homologies to their potential orthologs found in NR database by BLASTP search (**Table 1**). When homologies among the *HaV53* paralogs and their orthologs in other organisms were compared, identities among *HaV53* paralogs were much higher than identities to orthologs, presumably suggesting that these redundancies were based on recent gene duplication rather than horizontal gene transfer from the species with the closest orthologs (Santini et al., 2013).

**Table 1:** Redundant genes observed in *HaV53* open reading frames (ORFs).

Gene group	ID % to paralogs	Predicted function	Closest orthologs	ID % to orthologs
ORF1, ORF3, ORF118, ORF182	95.2 ~ 96.5	Transposases	Bacterial proteins	24.31 ~ 35.29
ORF9, ORF162	48.32	Glycosyltransferase,	Marseillevirus NCVOG2757	24.79 ~ 26.16
ORF28, ORF29	100	VV32-like packaging_ATPase	OtV1 NCVOG0249	48.89 ~ 49.07
ORF53, ORF183	95.35	Uncharacterized_protein	MpV1 NCVOG5117	18.05 ~ 38.51
ORF202, ORF214,	88.6	Outer membrane protein	Bacterial proteins	17.66 ~ 19.34
ORF203, 204, 205	70.3 ~ 78.9	Putative glutamine rich 2-like protein	Eukaryote proteins	23.00 ~ 24.75


*Redundant genes, or paralogs, in *HaV53*, presumably products of gene duplications, were identified by all-to-all BLASTP using *HaV53* ORFs as query and database.*

To certify that *HaV53* is indeed a phycodnavirus, we conducted phylogenetic analysis of DNA polymerase B, capsid protein, and D5-like helicase primase, and found that *HaV53* genes cluster with their orthologs from other phycodnaviruses, confirming that *HaV53* is a new member of the family (**Supplementary Figure S2**).

### Similarity of *HaV53* to Other NCLDVs

**FIGURE 2:**
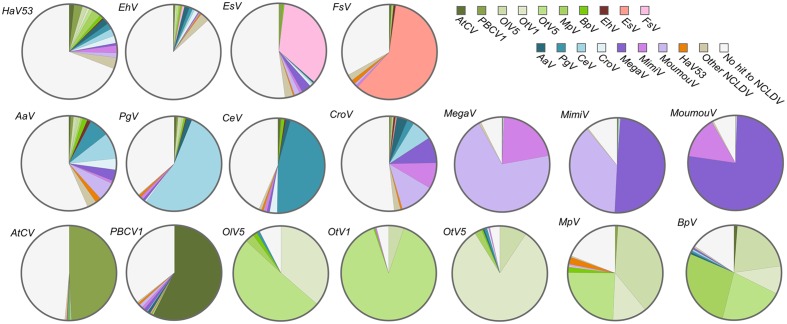
**Source viral species of the best-matching nucleocytoplasmic large DNA viruses (NCLDV) orthologs for genes from *HaV53*, Megaviridae, and *Phycodnaviridae*.** The best-hit homologs in the NCLDV database, to viral ORFs were determined by BLASTP (*E*-value < 10^-5^), and source NCLDVs were identified.

To further evaluate the relatedness of *HaV53* and other NCLDVs, we first conducted a blanket comparison of all the HaV53 ORFs with NCLDV genes. To this end, we constructed an NCLDV protein sequence database consisting of all the proteins encoded by representative, fully sequenced and annotated NCLDVs, including Megaviridae, *Phycodnaviridae, Marseilleviridae*, *Ascoviridae*, *Asfarviridae*, *Baculoviridae*, *Herpesviridae*, *Iridoviridae*, *Poxviridae*, Pandraviruses, and Pithovirus. First, we identified the NCLDV orthologs of each gene in *HaV53*, and identified the source viral species of the best-hit target genes (**Figure 2**). For comparison, the same analyses were conducted for genes carried by 17 members of *Phycodnaviridae* and proposed Megaviridae (**Figure 2**). Each virus showed a characteristic pattern in the distribution of best-hit sources. As expected, three lineages of mimiviruses, *MimiV* (lineage A), *MoumouV* (lineage B), and MegaV (lineage C), were found to be significantly related by this analysis; about 88, 92, and 92%, of *MimiV*, *MoumouV*, and MegaV genes, respectively, were most homologous to the other two mimivirus lineages. Among the proposed Megaviridae with smaller genome sizes, AaV and *CroV* exhibited similarities to both mimiviruses and smaller Megaviridae, while PgV and CeV showed significant relatedness to each other. AaV, PgV, and CeV are currently classified as *Phycodnaviridae*, although they did not show strong similarities to the other members of the family. A group including chloroviruses, *OtV1/5*, OlV5, *MpV*, and BpV showed large proportions of orthologs identified from the group. *EhV* did not show significant similarities to any NCLDV, and contained the smallest proportion of genes (16.5%) showing similarities to NCLDVs. As expected, two phaeoviruses, *EsV* and FsV, showed significant similarities to each other. *HaV53* genes showed a similar degree of similarity to both Megaviridae and *Phycodnaviridae* minus Megaviridae, with 14.0 and 12.8%, respectively. These results indicate that members of *Phycodnaviridae* exhibit homologies to particular group of other family members, or showed low homologies to others. These observation implies that *Phycodnaviridae* comprise several cluster of the members, which are not necessarily homologous to each other.

**FIGURE 3:**
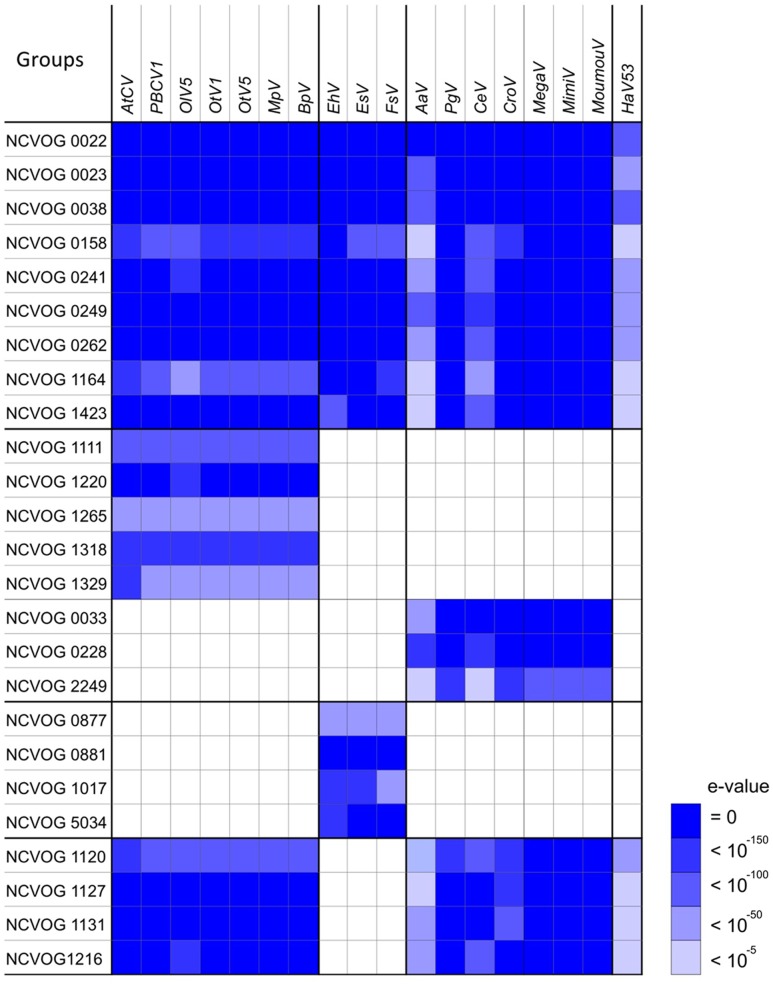
**Nucleo-Cytoplasmic Virus Orthologous Groups (NCVOG) orthologs in *Phycodnaviridae* and *Mimiviridae*.** The NCVOG orthologs in each virus were identified by BLASTP search (*E*-value < 10^-5^), and similarities between the query viral factors and NCVOGs are displayed. When several different viral ORFs hit an NCVOG, the viral factor with the highest bit score was chosen.

We next explored the presence/absence of all NCVOGs in the 18 viruses. We searched for NCVOG homologs for each viral ORF with size 100 aa or larger using BLASTP (*E*-value < 10^-5^), then identified the target sequence that gave the highest bit score (**Figure 3**). When more than one viral ORF hit the same NCVOG, the ORF that gave the highest bit score was identified as the NCVOG ortholog. Nine NCVOGs were found in all the analyzed viruses including *HaV53* (**Figure 3**): NCLDV major capsid protein (NCVOG0022), D5-like helicase primase (NCVOG0023), DNA polymerase B (NCVOG0038), an uncharacterized protein (NCVOG0158), proliferating cell nuclear antigen protein (NCVOG0241), A32-type packaging ATPase (NCVOG0249), poxvirus late transcription factor VLTF3-like protein (NCVOG0262), A1L transcription factor/late transcription factor VLTF2 (NCVOG1164), and a protein with uncharacterized C-terminal domain conserved in iridovirus, phycodnavirus, and mimivirus (NCVOG1423). The homologies among the orthologs were further confirmed by reciprocal BLASTP searches. For three orthologs, NCVOG0038, NCVOG0249, and NCVOG0262, all combinations identified from the eighteen viruses showed significant similarities to each other (*E*-value < 10^-5^), and all combinations for NCVOG1423 showed significant similarities except CeV and *EhV*. The other five orthologs showed similarities for some of the analyzed combinations (**Supplementary Figures S3A–E**). Among them, NCVOG0023 coded by Megaviridae and the rest of *Phycodnaviridae* did not show similarities, showing clear segregation between the groups (**Supplementary Figure S3B**). Significant similarities were observed between all the combinations of members of each group (**Supplementary Figure S3B**). The *HaV53* NCVOG0023 showed similarity with those of Megaviridae (**Supplementary Figure S3B**). At the same time, NCVOG0023 phylogenetic analysis revealed less clear segregation of *Phycodnaviridae* and *Mimiviridae* members from other NCLDVs (**Supplementary Figure S1C**), suggesting that NCVOG0023 may not be suitable to be used as a hallmark gene for classification.

### Phylogenetic/Phylogenomic Positions of *HaV53* among Phycodnaviridae and Megaviridae Members

**FIGURE 4:**
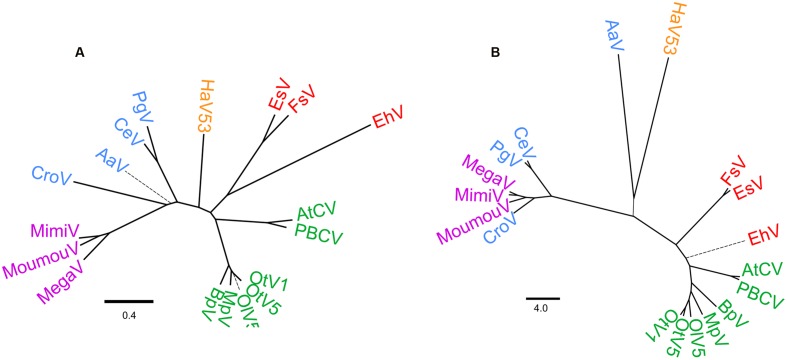
**Phylogenetic relationships of *Phycodnaviridae* and Megaviridae.** **(A)** Phylogenetic tree based on the concatenated nine core gene-encoded protein sequences. **(B)** Dendrogram based on PGAP analysis. Branches with bootstrap value < 95% are shown as thick solid lines, <75% as thin solid lines, and <50% as thin broken lines.

Based on the set of common orthologs identified above, we next analyzed phyletic relationships between the 18 viruses (**Figure 4**). As previously reported, three viruses currently classified as phycodnaviruses, AaV, PgV, and CeV, associate with mimivirus and *CroV* (Santini et al., 2013; Moniruzzaman et al., 2014), segregating from the remaining *Phycodnaviridae* and *HaV53*, with some ambiguity concerning the position of AaV (**Figure 4A**). The rest of *Phycodnaviridae* clustered into three distinct groups: one with *EhV*, *EsV*, and FsV; *HaV53*; and the seven remaining viruses including *PBCV* (**Figure 4A**).

Further, we analyzed phyletic relations by pan-genome analysis (**Figure 4B**). This approach allows us to evaluate phylogenomic relationships among the dataset by *de novo* clustering, and is thus independent of the NCVOG database. Other than AaV and *HaV53* being clustered with low confidence, overall taxonomic/phylogenetic relationships were coherent with the results based on phylogenetic analysis (**Figures 4A,B**). Results of these two analyses revealed that current *Phycodnaviridae* can be categorized into four groups. The first group includes *AtCV*, *PBCV*, OlV5, *OtV1*, *OtV5*, *MpV*, and BpV. The second group consists of *EhV*, *EsV*, and FsV. The third group includes *Phycodnavidae* members that belong to Megaviridae, with some ambiguity concerning the position of AaV. *HaV53* appears to be independent of the other three groups, positioned between the *EhV* group and the Megaviridae-phycodnaviruses.

As shown in **Figure 3**, we also identified several NCVOGs that characteristically exist in specific viral groups deduced from the phylogenetic/pan-genomic analysis (**Figure 4**). For example, five NCVOGs with unknown functions (NCVOG1111, 1220, 1265, 1318, and 1329) were found in the *PBCV* group, but not in others. Also, four NCVOGs, unknown functions (NCVOG0877, 0881, 1017, and 5034) were found in *EhV*, *EsV*, and FsV exclusively, but not in other groups. In contrast, metallopeptidase WLM (NCVOG1120), transcription initiation factor IIB (NCVOG1127), and two uncharacterized proteins (NCVOG1131 and 1216) exist in all the analyzed viruses but *EhV*, *EsV*, and FsV. Some of the previously identified Megaviridae hallmark genes, DNA topoisomerase I (NCVOG0033), ATP-dependent protease (NCVOG0228), and DNA-directed RNA polymerase II subunit RPB7 (NCVOG2249), were found in the proposed Megaviridae including mimiviruses. Further, homologies among these orthologs were analyzed, and the orthologs that showed significant similarities between all combinations of species are summarized in **Table 2**.

**Table 2:** Group hallmark genes.

Group	Nucleo-Cytoplasmic Virus Orthologous Groups (NCVOG)	Descriptions
*PBCV*-group	NCVOG1318	Uncharacterized protein
	NCVOG1329	Uncharacterized protein
*EhV*-group	NCVOG0881	Uncharacterized protein
	NCVOG1017	Uncharacterized protein
‘Megaviridae’	NCVOG0033	DNA topoisomerase I
	NCVOG0228	ATP-dependent protease
	NCVOG0105	MutS (for MoumouV, NCVOG0199)


**FIGURE 5:**
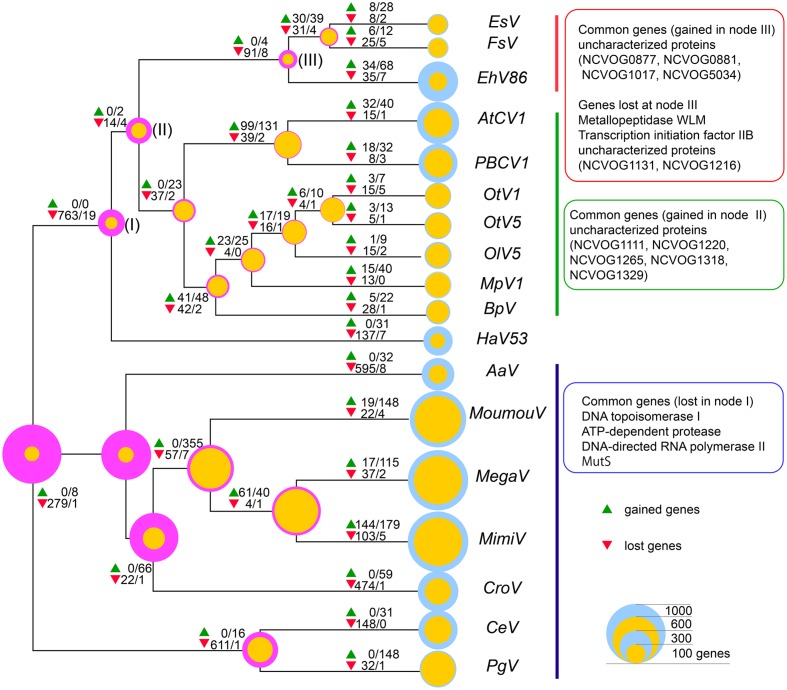
**Inferred gene gain/loss patterns and NCLDV hallmark genes.** Numbers of genes gained (green triangles) and lost (red triangles) inferred using COUNT implementing Wagner parsimony are indicated at each node. The former and the latter numbers, separated by slashes, indicated at the symbols are numbers of gained or lost genes inferred by the analysis with gain penalties 5 and 1, respectively. Magenta and yellow circles at the nodes indicate inferred NCVOGs numbers with gain penalties 5 and 1, respectively, and blue circles indicate numbers of genes of each analyzed virus. Sizes of the circles represent numbers of genes.

*NCVOG specifically associated with the groups of viruses were identified (**Figure 5**), and the genes that showed significant homologies by comparisons of all the combinations viruses in the group are listed.*

One of the previously identified hallmark genes (Ogata et al., 2011), MutS7 (NCVOG0105), was not found in *MoumouV* by this analysis. However, *MoumouV* does possess MutS (YP_007354438.1), while it is categorized as NCVOG0199 by this method. The protein possesses MutS7 features including MutS domains II, III, and IV, and the orthologs in Megaviridae, including *MoumouV* NCVOG0199, exhibit significant similarities in all combinations, while being absent in other *Phycodnaviridae* (**Table 2**; **Supplementary Figure S4A**). Furthermore, asparagine synthetase (NCVOG0061) and polyA polymerase (NCVOG0575) genes were not found in AaV (**Supplementary Figures S4B,C**). With consistently higher e-values for other Megaviridae hallmark genes, AaV could be defined as an outlier of Megaviridae. In addition, NCVOG0061 orthologs were also found in several *PBCV*-type *Phycodnaviridae* members, and these displayed sequence similarities with those in Megaviridae members, casting questions on its status as a hallmark gene (Moniruzzaman et al., 2014) (**Supplementary Figure S4B**).

Finally, evolutionary scenarios of viruses were reconstructed using COUNT software (Csuros, 2010; Kamneva and Ward, 2014) with the core gene phylogenetic tree as the guide tree topology (**Figure 5**). By this program, the size of the common ancestor virus and subsequent evolutional process can be inferred assuming different pressures for acquisitions and loss of genes, using different gene gain/loss penalties. When lower penalty for gene gains that gives the ancestor with only sixty-one NCVOG genes was chosen, *Mimiviridae* members were presumed to go through major gene gain when diverged from the smaller *Megaviridae* to reach to contemporary genome sizes. On the other hand, when high penalty for gene gain that gives the common ancestor with 996 NCVOGs was adopted, massive gene reduction at the timing of the divergence of the ancestor of *Phycodnaviridae* minus Megaviridae group from the ancestor of Megaviridae was inferred. The nodes where the hallmark genes identified in **Figure 3** emerged or were lost were also predicted by the analysis (**Figure 5**). The inferred gene gain/loss ratio and estimated numbers of gene gains/losses per COG varied widely between clades as reported previously by analyzing evolutionally processes of closely related mimiviruses and phycodnaviruses (Filee, 2015).

## Discussion

The present study reveals that *HaV53*, the first raphidovirus isolated and characterized (Nagasaki and Yamaguchi, 1997; Nagasaki et al., 2005), is a unique NCLDV in many respects. While ∼31% of *HaV53* genes display homology to NCLDV proteins, ∼11% show homology to bacterial and eukaryotic proteins, and ∼58% show no homology to any proteins identified to date. That a significant percentage of *HaV53* genes do not show homology with proteins in the databases is typical of viruses belonging to a newly characterized lineage with no other sequenced representatives. Gene duplications followed by mutations or genetic drift and horizontal gene transfer from host to virus are presumable sources of unique *HaV53* genes. By a heuristic approach, several *HaV53* ORFs were identified as potential results of gene duplication (**Table 1**). On the other hand, possibility of horizontal gene transfer cannot be investigated, at this point, because of the scarcity of *H. akashiwo* genome/transcriptome information obtained to date.

*HaV53* possesses 74 genes exhibiting homology to NCVOGs (**Supplementary Table S1**), while its composition is unique relative to other members of *Phycodnaviridae* or proposed Megaviridae (**Figures 1** and **2**). For example, *HaV53* does not possess the Megaviridae hallmark genes MuTS7, DNA-directed RNA polymerases, polyA polymerase, and DNA topoisomerase I (Ogata et al., 2011; Santini et al., 2013; Moniruzzaman et al., 2014), supporting the conclusion that *HaV53* is not likely a member of the proposed family. Notably, *HaV53*_ORF179 exhibits significant homology to bacterial asparagine synthetase A with an aminoacyl-transfer RNA synthetase domain (WP_003512022.1). Some mimiviruses characteristically possess aspartyl/asparaginyl-tRNA synthetase (Yutin et al., 2014). In the case of *HaV53*_ORF179, however, the motif corresponding to the anti-codon binding domain is missing, suggesting that the protein may not exhibit tRNA synthase activity. Several orthologs that are shared among members of the *PBCV* and *EhV* groups are not found in *HaV53* (**Figure 3**). These data underscore the uniqueness of *HaV53* among *Phycodnaviridae* and proposed Megaviridae.

Importantly, *HaV53* does not possess DNA-directed RNA polymerase or polyA RNA polymerase, indicating that *HaV53* depends on its host’s transcription machinery. On the other hand, as observed in many different NCLDVs, *HaV53* harbors several genes related to regulation of transcription, including transcription initiation factors, mRNA capping enzyme subunits, and ribonuclease III.

Among the viruses analyzed in this study, AaV, *EhV*, and *HaV53* possess higher proportions of unique genes that are not homologous to other NCLDV genes (**Figures 2** and **5**). This may be the main reason for the less well-defined phyletic positions of these three viruses in the results of pan-genomic analysis (**Figure 4B**). In particular, AaV has been characterized as a Megaviridae-type phycodnavirus (Moniruzzaman et al., 2014). However, NCVOG orthologs commonly found in Megaviridae-type phycodnaviruses exhibit low homology to the corresponding genes in AaV (**Figure 4**). In addition, polyA polymerase (**Supplementary Figure S4C**) and asparagine synthetase (Moniruzzaman et al., 2014) are missing exclusively in AaV. These observations and **Figure 4** show that AaV may be a non-standard member, or rather, outlier of the Megaviridae. AaV and *HaV53* clustered closely, though with low confidence, in our pan-genomic analysis. We also directly compared AaV and *HaV53* genes by all-to-all BLASTP, and consistent with the results presented in **Figure 2**, they did not exhibit particularly high homology to each other.

We observe a segregation of viruses currently considered to be phycodnaviruses into at least four groups. The proposed Megaviridae-phycodnavirus group segregates from the rest. In addition, the *EhV* group clearly segregates from other *Phycodnaviridae*, consistent with the argument of Allen et al. (2006a; 2006b). *HaV53* does not show a strong association with any of the three groups, and thus presumably represents a novel, independent group. Accordingly, we found several orthologs that specifically associate with each group of *Phycodnaviridae* (**Table 2**). These group-specific genes can be utilized as hallmarks to classify *Phycodnaviridae* in the future.

Currently, there were two major scenarios for evolution of Giant dsDNA viruses; the ‘reduction model’ and the ‘expansion model.’ The ‘reduction model’ is based on the idea that the viruses presumably emerged from much more complex organisms with larger sizes of genome, and reached to current status by genome simplifications (Raoult et al., 2004; Martin et al., 2010; Boyer et al., 2011; Nasir and Caetano-Anollés, 2015). In the ‘expansion model,’ the viruses are presumed to descend from common ancestor virus with much smaller genomes, and reaching to contemporary sizes and diversity by progressively acquiring genes (Yutin et al., 2014). Assuming distinctive gene gain/loss penalty scores to yield ancestor virus with distinctive NCVOG numbers, two evolutional paths resulting from the contrasting models were reproduced (**Figure 5**). When the ‘reduction model’ was reproduced with high gene gain penalty, the massive gene losses during the early stage of divergence of *Phycodnaviridae* minus Megaviridae from the rest (i.e., Megaviridae) were inferred [**Figure 5**, at node (I)]. On contrary, according to the ‘expansion model’ inferred by using lower gain penalty, major gene gains were observed after *Mimiviridae* diverged from smaller members of proposed Megaviridae. Comparative genome analyses of closely related members of *Phycodnaviridae* and *Mimiviridae* revealed specific patterns of gene gains and losses during the divergence of the lineages (Filee, 2015). Such future studies comprise of more distant viruses, possibly with more lineages, will provide insights into the overall evolutionally process of the Giant dsDNA viruses.

As *Phycodnaviridae* encompasses viruses infecting hosts of such vast diversity, they are expected to adopt varied strategies, and thus to develop genomes coding for distinctive genes with an array of functions during evolution. Our results, along with several previous observations, strongly suggest that classification of *Phycodnaviridae* does not represent current similarity in their genetic components, viral life cycle, and evolutionary relatedness. Systematic reclassification of the family based on current knowledge may not only provide better taxonomy of viruses but also lead to a better representation and understanding of evolution of NCLDVs, which remain enigmatic biological entities.

## Author Contributions

FM conducted data analysis and participated in discussion and writing the paper. SU designed research, conducted data analysis, and wrote the paper.

## Conflict of Interest Statement

The authors declare that the research was conducted in the absence of any commercial or financial relationships that could be construed as a potential conflict of interest.

## References

[B1] Abergel C. Legendre M. Claverie J. M. (2015). The rapidly expanding universe of giant viruses: *Mimivirus*, *Pandoravirus*, *Pithovirus* and *Mollivirus*. *FEMS Microbiol. Rev.* 39 779–796. 10.1093/femsre/fuv037 26391910

[B2] Abrahao J. S. Dornas F. P. Silva L. C. Almeida G. M. Boratto P. V. Colson P. (2014). *Acanthamoeba polyphaga* mimivirus and other giant viruses: an open field to outstanding discoveries. *Virol. J.* 11:120 10.1186/1743-422X-11-120 PMC4083134 24976356

[B3] Allen M. J. Schroeder D. C. Donkin A. Crawfurd K. J. Wilson W. H. (2006a). Genome comparison of two Coccolithoviruses. *Virol. J.* 3:15 10.1186/1743-422x-3-15 PMC1440845 16553948

[B4] Allen M. J. Schroeder D. C. Holden M. T. Wilson W. H. (2006b). Evolutionary history of the Coccolithoviridae. *Mol. Biol. Evol.* 23 86–92. 10.1093/molbev/msj010 16151186

[B5] Arslan D. Legendre M. Seltzer V. Abergel C. Claverie J. M. (2011). Distant *Mimivirus* relative with a larger genome highlights the fundamental features of Megaviridae. *Proc. Natl. Acad. Sci. U.S.A.* 108 17486–17491. 10.1073/pnas.1110889108 21987820 PMC3198346

[B6] Baudoux A. C. Brussaard C. P. (2005). Characterization of different viruses infecting the marine harmful algal bloom species *Phaeocystis globosa*. *Virology* 341 80–90. 10.1016/j.virol.2005.07.002 16081120

[B7] Boyer M. Azza S. Barrassi L. Klose T. Campocasso A. Pagnier I. (2011). *Mimivirus* shows dramatic genome reduction after intraamoebal culture. *Proc. Natl. Acad. Sci. U.S.A.* 108 10296–10301. 10.1073/pnas.1101118108 21646533 PMC3121840

[B8] Brussaard C. P. Short S. M. Frederickson C. M. Suttle C. A. (2004). Isolation and phylogenetic analysis of novel viruses infecting the phytoplankton *Phaeocystis globosa* (Prymnesiophyceae). *Appl. Environ. Microbiol.* 70 k3700–3705. 10.1128/AEM.70.6.3700-3705.2004 PMC427783 15184176

[B9] Chang F. H. Anderson C. Boustead N. C. (1990). First record of a Heterosigma (Raphidophyceae) bloom with associated mortality of cage-reared salmon in Big Glory Bay, New-Zealand. *N. Z. J. Mar. Freshwater Res.* 24 461–469. 10.1080/00288330.1990.9516437

[B10] Chelikani V. Ranjan T. Zade A. Shukla A. Kondabagil K. (2014). Genome segregation and packaging machinery in *Acanthamoeba polyphaga* mimivirus is reminiscent of bacterial apparatus. *J. Virol.* 88 6069–6075. 10.1128/JVI.03199-13 24623441 PMC4093880

[B11] Chen J. Cassar S. C. Zhang D. Gopalakrishnan M. (2005). A novel potassium channel encoded by *Ectocarpus siliculosus* virus. *Biochem. Biophys. Res. Commun.* 326 887–893. 10.1016/j.bbrc.2004.11.125 15607752

[B12] Claverie J. M. Abergel C. (2010). *Mimivirus*: the emerging paradox of quasi-autonomous viruses. *Trends Genet.* 26 431–437. 10.1016/j.tig.2010.07.003 20696492

[B13] Claverie J. M. Abergel C. Ogata H. (2009). *Mimivirus*. *Curr. Top. Microbiol. Immunol.* 328 89–121. 19216436 10.1007/978-3-540-68618-7_3

[B14] Claverie J. M. Ogata H. Audic S. Abergel C. Suhre K. Fournier P. E. (2006). *Mimivirus* and the emerging concept of “giant” virus. *Virus Res.* 117 133–144. 10.1016/j.virusres.2006.01.008 16469402

[B15] Colson P. Yutin N. Shabalina S. A. Robert C. Fournous G. La Scola B. (2011). Viruses with more than 1,000 genes: *Mamavirus*, a new *Acanthamoeba polyphaga* mimivirus strain, and reannotation of *Mimivirus* genes. *Genome Biol. Evol.* 3 737–742. 10.1093/gbe/evr048 21705471 PMC3163472

[B16] Csuros M. (2010). Count: evolutionary analysis of phylogenetic profiles with parsimony and likelihood. *Bioinformatics* 26 1910–1912. 10.1093/bioinformatics/btq315 20551134

[B17] Filee J. (2015). Genomic comparison of closely related Giant Viruses supports an accordion-like model of evolution. *Front. Microbiol.* 6:593 10.3389/fmicb.2015.00593 PMC4468942 26136734

[B18] Fischer M. G. Allen M. J. Wilson W. H. Suttle C. A. (2010). Giant virus with a remarkable complement of genes infects marine zooplankton. *Proc. Natl. Acad. Sci. U.S.A.* 107 19508–19513. 10.1073/pnas.1007615107 20974979 PMC2984142

[B19] Fischer W. B. Sansom M. S. (2002). Viral ion channels: structure and function. *Biochim. Biophys. Acta* 1561 27–45. 10.1016/S0304-4157(01)00009-0 11988179

[B20] Frohns F. Kasmann A. Kramer D. Schafer B. Mehmel M. Kang M. (2006). Potassium ion channels of *Chlorella* viruses cause rapid depolarization of host cells during infection. *J. Virol.* 80 2437–2444. 10.1128/jvi.80.5.2437-2444.2006 16474150 PMC1395400

[B21] Gazzarrini S. Severino M. Lombardi M. Morandi M. DiFrancesco D. Van Etten J. L. (2003). The viral potassium channel Kcv: structural and functional features. *FEBS Lett.* 552 12–16. 10.1016/S0014-5793(03)00777-4 12972145

[B22] Hamacher K. Greiner T. Ogata H. Van Etten J. L. Gebhardt M. Villarreal L. P. (2012). Phycodnavirus potassium ion channel proteins question the virus molecular piracy hypothesis. *PLoS ONE* 7:e38826 10.1371/journal.pone.0038826 PMC3369850 22685610

[B23] Honjo T. (1993). “Overview on bloom dynamics and physiological ecology of *Heterosigma akashiwo*,” in *Toxic Phytoplankton Blooms in the Sea*, eds Smayda T. J. Shimizu Y. (Amsterdam: Elsevier), 33–41.

[B24] Iyer L. M. Balaji S. Koonin E. V. Aravind L. (2006). Evolutionary genomics of nucleo-cytoplasmic large DNA viruses. *Virus Res.* 117 156–184. 10.1016/j.virusres.2006.01.009 16494962

[B25] Kamneva O. K. Ward N. L. (2014). Reconciliation approaches to determining HGT, duplications, and losses in gene trees. *Methods Microbiol.* 41 183–199. 10.1016/bs.mim.2014.08.004

[B26] Koonin E. V. Yutin N. (2010). Origin and evolution of eukaryotic large nucleo-cytoplasmic DNA viruses. *Intervirology* 53 284–292. 10.1159/000312913 20551680 PMC2895762

[B27] Kuznetsov Y. G. Klose T. Rossmann M. McPherson A. (2013). Morphogenesis of mimivirus and its viral factories: an atomic force microscopy study of infected cells. *J. Virol.* 87 11200–11213. 10.1128/JVI.01372-13 23926353 PMC3807284

[B28] Lackey J. B. Lackey E. (1963). Microscopic algae and protozoa in the waters near Plymouth in August 1962. *J. Mar. Biol. Assoc. U.K.* 43 797–805. 10.1017/S0025315400025698

[B29] Larkin M. A. Blackshields G. Brown N. P. Chenna R. McGettigan P. A. McWilliam H. (2007). Clustal W and Clustal X version 2.0. *Bioinformatics* 23 2947–2948. 10.1093/bioinformatics/btm404 17846036

[B30] Legendre M. Lartigue A. Bertaux L. Jeudy S. Bartoli J. Lescot M. (2015). In-depth study of *Mollivirus sibericum*, a new 30,000-y-old giant virus infecting *Acanthamoeba*. *Proc. Natl. Acad. Sci. U.S.A.* 112 E5327–E5335. 10.1073/pnas.1510795112 26351664 PMC4586845

[B31] Mackenzie L. (1991). Toxic and noxious phytoplankton in Big Glory Bay, Stewart-Island, New-Zealand. *J. Appl. Phycol.* 3 19–34. 10.1007/Bf00003916

[B32] Martin D. P. Boyer M. Madoui M.-A. Gimenez G. La Scola B. Raoult D. (2010). Phylogenetic and phyletic studies of informational genes in genomes highlight existence of a 4th domain of life including giant viruses. *PLoS ONE* 5:e15530 10.1371/journal.pone.0015530 PMC2996410 21151962

[B33] Monier A. Larsen J. B. Sandaa R. A. Bratbak G. Claverie J. M. Ogata H. (2008). Marine mimivirus relatives are probably large algal viruses. *Virol. J.* 5:12 10.1186/1743-422X-5-12 PMC2245910 18215256

[B34] Moniruzzaman M. LeCleir G. R. Brown C. M. Gobler C. J. Bidle K. D. Wilson W. H. (2014). Genome of brown tide virus (AaV), the little giant of the Megaviridae, elucidates NCLDV genome expansion and host-virus coevolution. *Virology* 46 60–70. 10.1016/j.virol.2014.06.031 25035289

[B35] Mutsafi Y. Shimoni E. Shimon A. Minsky A. (2013). Membrane assembly during the infection cycle of the giant *Mimivirus*. *PLoS Pathog.* 9:e1003367 10.1371/journal.ppat.1003367 PMC3667779 23737745

[B36] Nagasaki K. Shirai Y. Tomaru Y. Nishida K. Pietrokovski S. (2005). Algal viruses with distinct intraspecies host specificities include identical intein elements. *Appl. Environ. Microbiol.* 71 3599–3607. 10.1128/Aem.71.7.3599-3607.2005 16000767 PMC1169056

[B37] Nagasaki K. Yamaguchi M. (1997). Isolation of a virus infectious to the harmful bloom causing microalga *Heterosigma akashiwo* (Raphidophyceae). *Aquat. Microb. Ecol.* 13 135–140. 10.3354/Ame013135

[B38] Nasir A. Caetano-Anollés G. (2015). A phylogenomic data-driven exploration of viral origins and evolution. *Sci. Adv.* 1:e1500527 10.1126/sciadv.1500527 PMC4643759 26601271

[B39] Ogata H. Ray J. Toyoda K. Sandaa R. A. Nagasaki K. Bratbak G. (2011). Two new subfamilies of DNA mismatch repair proteins (MutS) specifically abundant in the marine environment. *ISME J.* 5 1143–1151. 10.1038/ismej.2010.210 21248859 PMC3146287

[B40] Ogura Y. Hayashi T. Ueki S. (2016). The complete genome sequence of a Phycodnavirus, *Heterosigma akashiwo* virus strain 53. *Genome Announc.* 4:e01279–16 10.1128/genomeA.01279-16 PMC5105112 27834719

[B41] O’Halloran C. Silver M. W. Holman T. R. Scholin C. A. (2006). *Heterosigma akashiwo* in central California waters. *Harmful Algae* 5 124–132. 10.1016/j.hal.2005.06.009

[B42] Raoult D. Audic S. Robert C. Abergel C. Renesto P. Ogata H. (2004). The 1.2-megabase genome sequence of *Mimivirus*. *Science* 306 1344–1350. 10.1126/science.1101485 15486256

[B43] Raoult D. Forterre P. (2008). Redefining viruses: lessons from *Mimivirus*. *Nat. Rev. Microbiol.* 6 315–319. 10.1038/nrmicro1858 18311164

[B44] Raoult D. La Scola B. Birtles R. (2007). The discovery and characterization of *Mimivirus*, the largest known virus and putative pneumonia agent. *Clin. Infect. Dis.* 45 95–102. 10.1086/518608 17554709

[B45] Rensel J. Homer R. A. Postel L. E. (1989). Effects of phytoplankton blooms on salmon aquaculture in Puget Sound, Washington: initial research. *Northwest Environ. J.* 5 53–59.

[B46] Rojas de Mendiola B. (1979). “Red tide dong the Peruvian coast,” in *Toxic Dinofagellate Bloom*, eds Taylor D. L. Seliger H. H. (Amsterdam: Elsevier), 183–190.

[B47] Santini S. Jeudy S. Bartoli J. Poirot O. Lescot M. Abergel C. (2013). Genome of *Phaeocystis globosa* virus PgV-16T highlights the common ancestry of the largest known DNA viruses infecting eukaryotes. *Proc. Natl. Acad. Sci. U.S.A.* 110 10800–10805. 10.1073/pnas.1303251110 23754393 PMC3696832

[B48] Schroeder D. C. Oke J. Malin G. Wilson W. H. (2002). *Coccolithovirus* (Phycodnaviridae): characterisation of a new large dsDNA algal virus that infects *Emiliania huxleyi*. *Arch. Virol.* 147 1685–1698. 10.1007/s00705-002-0841-3 12209309

[B49] Sharma V. Colson P. Pontarotti P. Raoult D. (2016). *Mimivirus* inaugurated in the 21st century the beginning of a reclassification of viruses. *Curr. Opin. Microbiol.* 31 16–24. 10.1016/j.mib.2015.12.010 26829206

[B50] Suzan-Monti M. La Scola B. Raoult D. (2006). Genomic and evolutionary aspects of *Mimivirus*. *Virus Res.* 117 145–155. 10.1016/j.virusres.2005.07.011 16181700

[B51] Taylor F. J. R. (1993). “Current problems with harmful phytoplankton blooms in British Colombia waters,” in *Toxic Phytoplankton Blooms in the Sea*, eds Smayda T. J. Shimizu Y. (Amsterdam: Elsevier), 699–703.

[B52] Thomas V. Bertelli C. Collyn F. Casson N. Telenti A. Goesmann A. (2011). Lausannevirus, a giant amoebal virus encoding histone doublets. *Environ. Microbiol.* 13 1454–1466. 10.1111/j.1462-2920.2011.02446.x 21392201

[B53] Tseng C. K. Zhou M. J. Zou J. Z. (1993). “Toxic phytoplankton studies in China,” in *Toxic Phytoplankton Blooms in the Sea*, eds Smayda T. J. Shimizu Y. (Amsterdam: Elsevier), 347–352.

[B54] Wilson W. H. Schroeder D. C. Allen M. J. Holden M. T. Parkhill J. Barrell B. G. (2005). Complete genome sequence and lytic phase transcription profile of a *Coccolithovirus*. *Science* 309 1090–1092. 10.1126/science.1113109 16099989

[B55] Yutin N. Colson P. Raoult D. Koonin E. V. (2013). Mimiviridae: clusters of orthologous genes, reconstruction of gene repertoire evolution and proposed expansion of the giant virus family. *Virol. J.* 10:106 10.1186/1743-422X-10-106 PMC3620924 23557328

[B56] Yutin N. Wolf Y. I. Koonin E. V. (2014). Origin of giant viruses from smaller DNA viruses not from a fourth domain of cellular life. *Virology* 46 38–52. 10.1016/j.virol.2014.06.032 PMC4325995 25042053

[B57] Yutin N. Wolf Y. I. Raoult D. Koonin E. V. (2009). Eukaryotic large nucleo-cytoplasmic DNA viruses: clusters of orthologous genes and reconstruction of viral genome evolution. *Virol. J.* 6:223 10.1186/1743-422X-6-223 PMC2806869 20017929

[B58] Zhao Y. Wu J. Yang J. Sun S. Xiao J. Yu J. (2012). PGAP: pan-genomes analysis pipeline. *Bioinformatics* 28 416–418. 10.1093/bioinformatics/btr655 22130594 PMC3268234

