# Supplementary material for: Evolution and Phylogeny of Large DNA Viruses, Mimiviridae and Phycodnaviridae Including Newly Characterized Heterosigma akashiwo Virus
Source: Front Microbiol. 2016 Nov 30;7:1942. doi: 10.3389/fmicb.2016.01942 (PMC5127864; doi:10.3389/fmicb.2016.01942)
Supplement: Supplementary file 1 [file Table_1.DOCX]

Supplemental Table 1 NCVOG orthologs of HaV53 genes.

| Genes | Ortholog definition | NCVOG ID | Ortholog in NCBI NR database |
| --- | --- | --- | --- |
| HaV53_ORF1 | transposase | NCVOG0321 |  |
| HaV53_ORF4 | uncharacterized protein | NCVOG0327 |  |
| HaV53_ORF9 | glycosyltransferase | NCVOG2757 |  |
| HaV53_ORF15 | DnaJ domain | NCVOG0046 | No ortholog in NR database |
| HaV53_ORF20 | Proliferating cell nuclear antigen | NCVOG0241 |  |
| HaV53_ORF21 | glycosyltransferase | NCVOG2123 |  |
| HaV53_ORF24 | uncharacterized protein | NCVOG1278 | No ortholog in NR database |
| HaV53_ORF26 | Poxvirus Late Transcription Factor VLTF3 like | NCVOG0262 |  |
| HaV53_ORF27 | uncharacterized protein | NCVOG0158 | *100% identity to *HaV01* UKCH-2 (BAB69883.1) |
| HaV53_ORF29 | packaging ATPase | NCVOG0249 | *95.25% identity *HaV01* AGB-1 (BAB69884.1) |
| HaV53_ORF30 | uncharacterized protein | NCVOG0780 |  |
| HaV53_ORF43 | TATA-box binding protein | NCVOG0313 |  |
| HaV53_ORF45 | RING finge containing E3 ubiquitin ligase | NCVOG0330 | Ubiquitin ligase, Nelumbo nucifera |
| HaV53_ORF47 | SWIB MDM2 domain containing protein | NCVOG1154 |  |
| HaV53_ORF48 | uncharacterized protein | NCVOG1278 |  |
| HaV53_ORF49 | Serine Threonine protein kinases | NCVOG0285 |  |
| HaV53_ORF51 | Cytidine and deoxycytidylate deaminase | NCVOG1064 |  |
| HaV53_ORF52 | uncharacterized protein | NCVOG5117 | Putative SH3-containing protein X2 |
| HaV53_ORF53 | uncharacterized protein | NCVOG1131 |  |
| HaV53_ORF54 | transcription initiation factor IIB | NCVOG1127 |  |
| HaV53_ORF57 | replication factor C small subunit 2 | NCVOG1351 | Putative replication factor C subunit 1 |
| HaV53_ORF61 | methyltransferase | NCVOG0564 |  |
| HaV53_ORF70 | uncharacterized protein | NCVOG5117 | Putative endosialidase |
| HaV53_ORF73 | Procollagen lysine2 oxoglutarate 5 dioxygenase | NCVOG1198 | Hypothetical protein |
| HaV53_ORF76 | DNA topoisomerase II | NCVOG0037 | Putative DNA topoisomerase II |
| HaV53_ORF77 | Adenine specific DNA methyltransferase | NCVOG0234 |  |
| HaV53_ORF78 | GIY YIG like endonuclease | NCVOG0062 |  |
| HaV53_ORF81 | KilA N domain protein | NCVOG1364 |  |
| HaV53_ORF90 | mRNA capping enzyme large subunit methyltransferase domain | NCVOG1117 | Putative mRNA cap guanine-N7 methyltransferase |
| HaV53_ORF95 | uncharacterized protein | NCVOG0632 |  |
| HaV53_ORF98 | mRNA capping enzyme N terminal ATPase and guanylyltransferase | NCVOG1451 | Putative mRNA capping enzyme |
| HaV53_ORF101 | A1L transcription factor late transcription factor VLTF 2 | NCVOG1164 |  |
| HaV53_ORF103 | uncharacterized C terminal domain conserved in irido asco phycodna mimi | NCVOG1423 |  |
| HaV53_ORF108 | Zn finger protein | NCVOG1343 |  |
| HaV53_ORF110 | uncharacterized protein | NCVOG4092 |  |
| HaV53_ORF111 | RuvC Holliday junction resolvases HJRs Poxvirus A22 family | NCVOG0278 |  |
| HaV53_ORF115 | putative integrase resolvase | NCVOG1175 |  |
| HaV53_ORF116 | putative integrase resolvase | NCVOG1175 |  |
| HaV53_ORF117 | transposase | NCVOG0321 | Putative Transcription regulator HTH |
| HaV53_ORF119 | uncharacterized protein | NCVOG1242 | Putative glycerophosphodiester phosphodiesterase |
| HaV53_ORF120 | Transcription factor S II TFIIS | NCVOG0272 | Putative transcription elongation factor S II |
| HaV53_ORF121 | YqaJ viral recombinase | NCVOG1192 | No ortholog in NR database |
| HaV53_ORF122 | FLAP like endonuclease XPG | NCVOG1060 | Putative XPG I-region family protein |
| HaV53_ORF125 | asparagine synthase | NCVOG0061 | Putative glutaminefructose-6-phosphate transaminase |
| HaV53_ORF127 | D5 like helicase primase | NCVOG0023 |  |
| HaV53_ORF128 | uncharacterized protein | NCVOG1137 |  |
| HaV53_ORF132 | unclassified DEAD SNF2 like helicases | NCVOG0031 |  |
| HaV53_ORF136 | uncharacterized protein | NCVOG1255 |  |
| HaV53_ORF137 | Ribonuclease III | NCVOG1354 |  |
| HaV53_ORF142 | uncharacterized protein | NCVOG5121 | Hypothetical protein |
| HaV53_ORF146 | short chain dehydrogenase | NCVOG1049 | Putative UDP-glucose 4-epimerase |
| HaV53_ORF147 | short chain dehydrogenase | NCVOG1049 | Putative gdp-d-mannose 4,6-dehydratase |
| HaV53_ORF150 | uncharacterized protein | NCVOG5117 |  |
| HaV53_ORF153 | FAD dependent thymidylate synthase | NCVOG1346 | Putative FAD-dependent thymidylate synthase |
| HaV53_ORF155 | MTG motif gene family protein | NCVOG5422 | Hypothetical protein |
| HaV53_ORF158 | uncharacterized domain DUF3627 | NCVOG1424 |  |
| HaV53_ORF160 | uncharacterized protein | NCVOG1023 |  |
| HaV53_ORF161 | glycosyltransferase | NCVOG2757 |  |
| HaV53_ORF169 | Patatin phospholipase | NCVOG0245 | Putative esterase |
| HaV53_ORF170 | metallopeptidase WLM | NCVOG1120 |  |
| HaV53_ORF177 | DNA polymerase elongation subunit family B | NCVOG0038 | *99.91% identity to *HaV01* B-family DNA polymerase (BAE06251.1) |
| HaV53_ORF181 | transposase | NCVOG0321 |  |
| HaV53_ORF182 | putative integrase resolvase | NCVOG1175 |  |
| HaV53_ORF183 | NCLDV major capsid protein | NCVOG0022 | *100% identity *HaV01* major capsid protein (BAB06835.1) |
| HaV53_ORF184 | Zn finger protein | NCVOG1343 | Hypothetical protein |
| HaV53_ORF186 | ALI motif family protein | NCVOG5421 |  |
| HaV53_ORF187 | DNA methylase | NCVOG4159 |  |
| HaV53_ORF194 | cytidine and deoxycytidylate deaminase | NCVOG1064 | Putative dCMP deaminase |
| HaV53_ORF195 | endonuclease DHD | NCVOG1074 | Hypothetical protein |
| HaV53_ORF221 | Proliferating cell nuclear antigen | NCVOG0241 | Hypothetical protein |
| HaV53_ORF228 | methyltransferase | NCVOG2648 |  |
| HaV53_ORF230 | exostosin | NCVOG0053 | Putative glycosyltransferase family 32 protein |
| HaV53_ORF236 | uncharacterized protein | NCVOG1312 | Hypothetical protein |
| HaV53_ORF238 | Esterase lipase superfamily | NCVOG0050 | Hypothetical protein, |

* Previously sequenced *HaV01* genes.
