# Supplementary figures and images for: Evolution and Phylogeny of Large DNA Viruses, Mimiviridae and Phycodnaviridae Including Newly Characterized Heterosigma akashiwo Virus
Source: Front Microbiol. 2016 Nov 30;7:1942. doi: 10.3389/fmicb.2016.01942 (PMC5127864; doi:10.3389/fmicb.2016.01942)

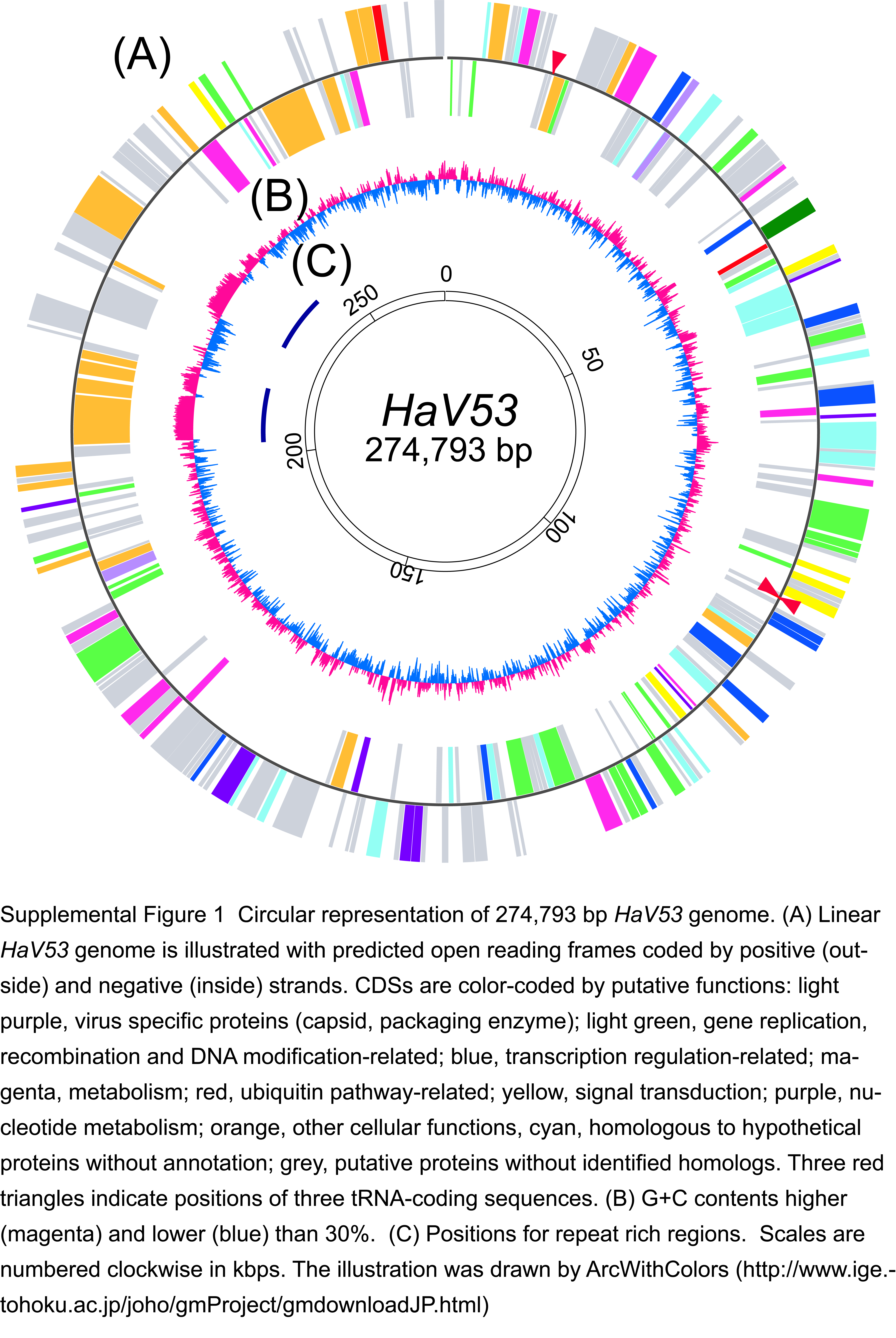

Supplement: Supplementary file 2 [file Image_1.TIF]

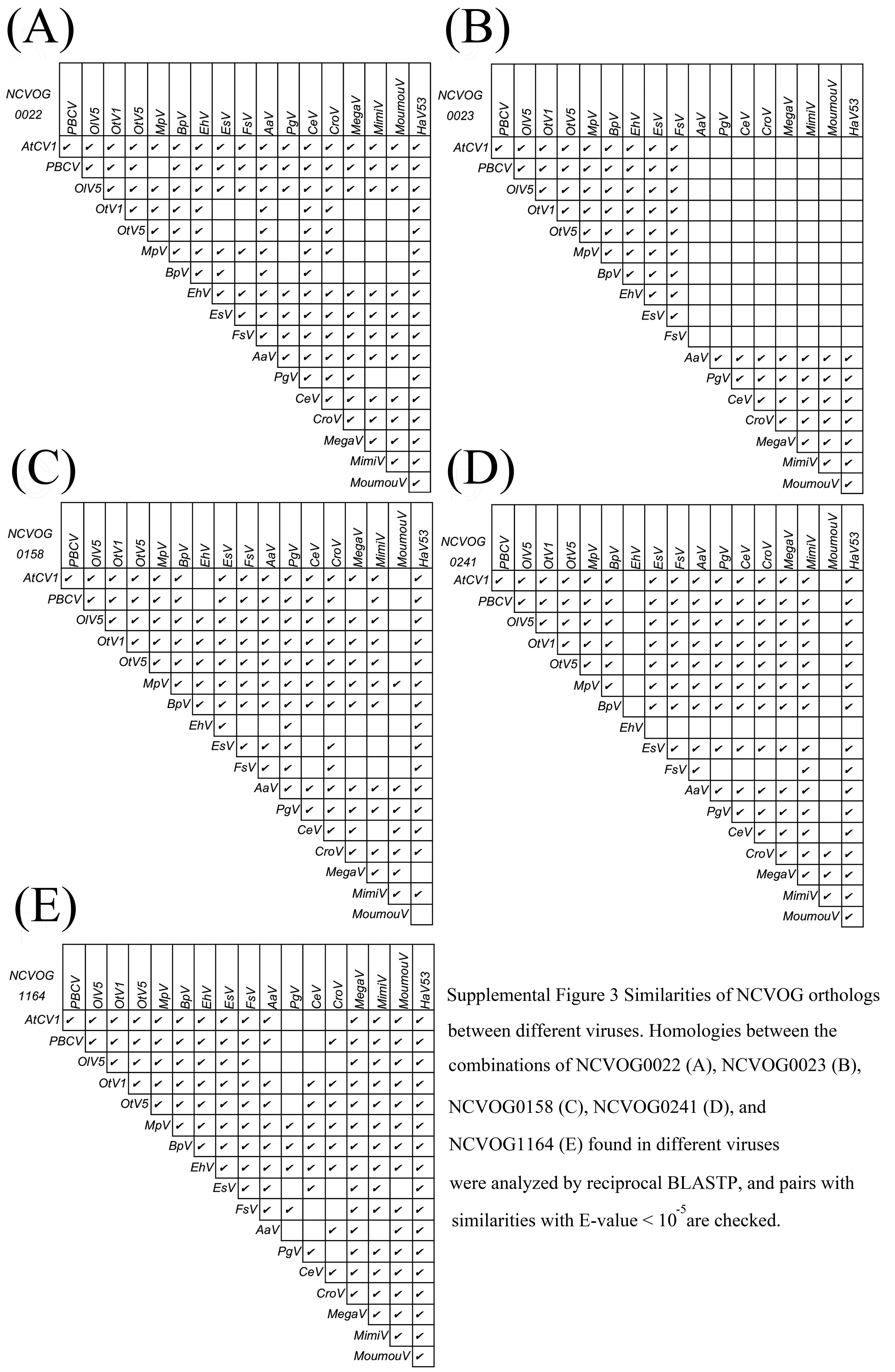

Supplement: Supplementary file 4 [file Image_3.TIF]

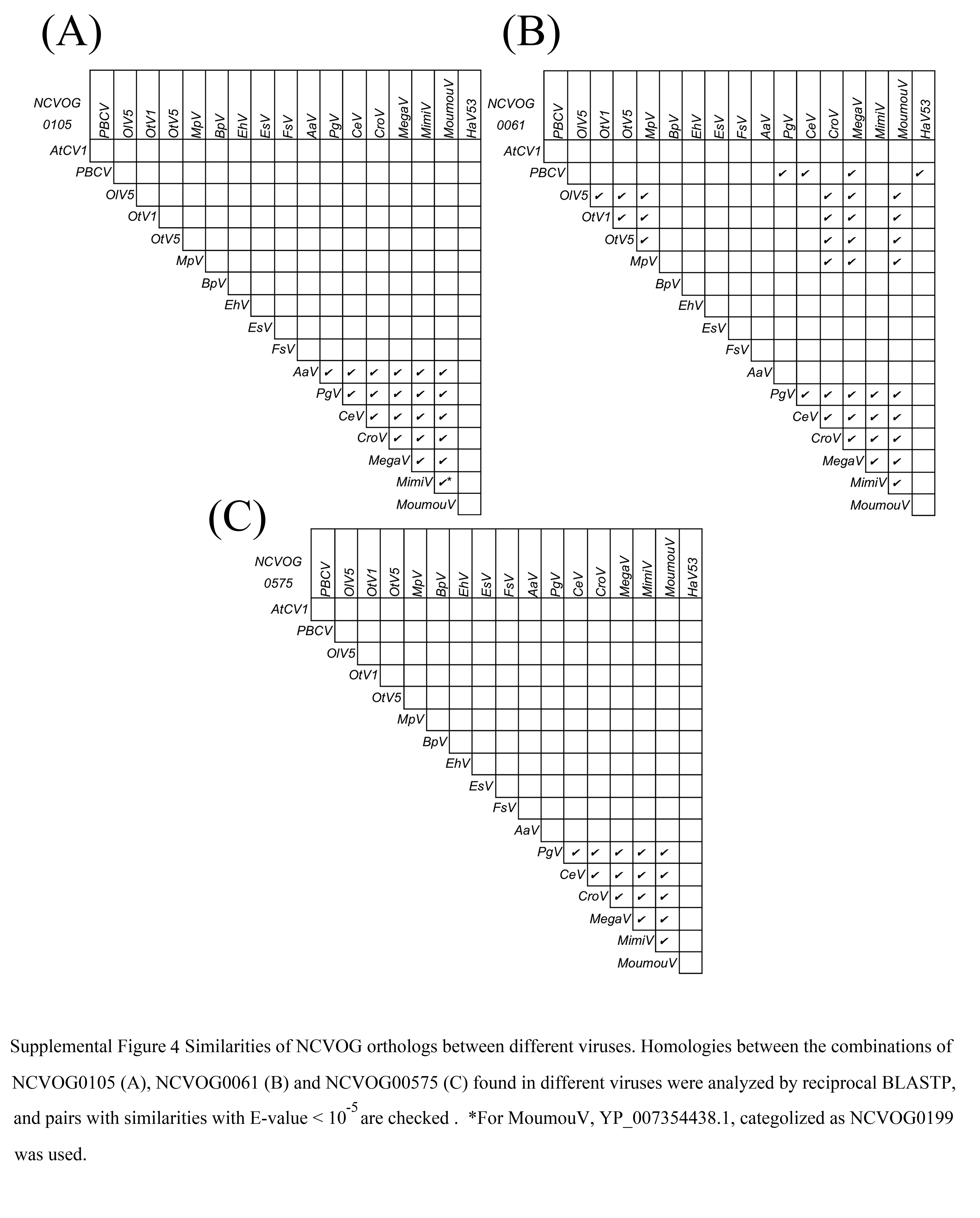

Supplement: Supplementary file 5 [file Image_4.TIF]
